# Supplementary material for: Modeling driver behavior in the dilemma zone based on stochastic model predictive control
Source: PLoS One. 2021 Feb 24;16(2):e0247453. doi: 10.1371/journal.pone.0247453 (PMC7904219; doi:10.1371/journal.pone.0247453)
Supplement: Attachment — Submitted filename: Response to Reviewers.docx [file pone.0247453.s002.docx]

**Response to Reviewer**

Dear Reviewers:

Manuscript ID: PONE-D-20-38386

Title: “Modeling Driver Behavior in the Dilemma Zone Based on Stochastic Model Predictive Control”

We wish to express our very deep appreciation, and the appreciation of all of us, to your great efforts and suggestions for our manuscript. They are valuable and very helpful for revising and improving our paper, as well as the important guiding to our researches.

The following is a point-to-point response to your comments and the responses are in red. The modification marked in red in revised version.

We tried our best to improve the manuscript and made some changes in the manuscript. These changes will not influence the content and framework of the paper. And here we did not list the changes but marked in red in revised paper. We appreciate for your warm work earnestly, and hope that the correction will meet with approval. Thank you for your time and patience. I look forward to receiving your letter.

Once again, we would like to thank you for the constructive comments and suggestions. Please feel free to contact us with any questions. We are looking forward to your reply.

Yours sincerely,

Authors

**Response to Reviewer #1**:

The research proposed a framework for driver behavior analysis in dilemma zone, which could provide the insights to the ability of drivers to perceive traffic conditions and the ability to plan the driving state. The modeling framework of driver behavior includes the perception module, decision-making module, and operation module. The perception module is used to model the ability of drivers to perceive traffic conditions and the ability to plan the driving state. The decision-making module is used to model the ability of the driver to realize the dynamic characteristics of vehicle motion and the ability to optimize the vehicle trajectory by evaluating the traffic condition and vehicle driving state. The operation module is used to model the response ability of the driver in the dilemma zone from cognitive to vehicle operation. The results show that the SMPC-based driver behavior model can effectively and accurately reflect the vehicle motion and dynamics under driving in the dilemma zone. This manuscript has potential of being published at PLOS ONE. I have some comments and suggestions for the authors to consider in their revision.

Response:

First of all, thanks for your comments and we believe these constructive comments will continuously improve our paper quality. Please find our detailed responses point-by-point as follows:

**Comment #1:** What would be data to calibrate this proposed model of driver behavior? I also would like to see how these types of models are applied in practice.

Response:

Thank you for your comments. We are sorry for not stating clearly about the data calibrating the proposed model. We would like to state the aim of this paper more clearly.

With the rapid increase in active safety systems and advanced driver assistances systems, building driving behaviors and skills by employing mathematical driver models have attracted great attention in recent research, and poses new challenges on the well-developed vehicle system dynamics. A thorough understanding of driver-vehicle-road interactions might enable development of safer and more comfortable road vehicles, along with furthering the development of V2I driving systems that improve the safety of drivers and adaptively of traffic signal system.

In general, driver has the ability of perceiving uncertainty and variety in driver-vehicle-road system, which may have a big impact on driver behavior. The main content of this paper is to set up the mathematical modeling method of driver behavior based on stochastic model predictive control, considering the driver’s internal abilities of driving surrounding perception, traffic condition estimation, and driving experiments learning, and the external abilities of operating vehicle under various driving scenarios. The proposed SMPC-based driver behavior model includes the perception, decision and execution modules, as to capture the effect of random variations of driver’s behavior in dilemma zone. The goal is to provide a feasible solution for modeling the driver behavior more accurately and then potentially improve the understanding of driver-vehicle-road system in the dilemma zone. Our aim is to explore the modeling framework of the driver behavior with moving horizon idea, and present a number of driver modeling methods to mimic various driving styles. Using vehicle dynamics simulation software CarSim which provided datasets that cover over 20 presentative vehicles, the characteristics of the proposed modeling methods are analyzed and discussed.

We added this explanation into the simulation and model verification section as:

In China, cars and SUVs make up the bulk of traffic flow in urban city. The Hatchback and SUV models in CarSim are closest to the actual vehicles running on the road. Therefore, we use the vehicle models of A-Class (Hatchback), B-Class (Hatchback), and D-Class (SUV) in the CarSim software package to verify the proposed model in this paper.

**Comment #2:** It seems that the model only considers a single vehicle or user. Could it be applied to multiple vehicles, as the intersection, the vehicles are in queen and interaction each other?

Response:

Thanks for your comments. Although we assume that when a driver approaches to the signalized intersection, he or she will perceive the traffic environment, including the traffic light, the distance from the stop-line and the traffic flow in the approaching direction.

This paper only focuses on model the signal driver and vehicle in the dilemma zone. Although in this paper, we make the assumption: When a driver approaching to the signalized intersection, he or she will perceive the traffic environment, including the traffic light, the distance from the stop-line and the traffic flow in the approaching direction, then he or she will estimate the traffic environment and driving state to decide whether to stop or proceed through the intersection. After the decision making, he or she optimizes the driving operation and operates the vehicle based on his or her driving experimental, physiological characteristics, psychological characteristics and so on. So the whole process can be divided into three modules: perception module, decision-making module, and operation module. The framework of the driving behavior is showed as Fig 1.

**Figure 1**. The framework of driving behavior in dilemma zone

In the assumption, we can use the driver perception and driver’s traffic state estimation to reflect the traffic status in front of vehicle. However, we have not verified the model under the environmental of multi-vehicles or traffic congestion as the reviewer mentioned. It needs to redesign the experimental processes with CarSim+Simulink in the simulator scene of hardware-in-the-loop and driver-in-the-loop. We will calibrate the model in such traffic conditions in further research.

**Comment #3:** The dilemma zone in the simulation is fixed zone or dynamic? If the yellow signal adjust according the motion of vehicle when it fall into the dilemma zone?

Response:

Thank you for your comments. In traffic event sequences, we use kinetic equations to define the range of dilemma zone and assume that:

When the driver realizes the yellow signal indication, the initial vehicle speed is $V_{0}$. If the driver decides to stop before the stop-line, the minimum distance to stop safely is[6]:

$$X_{0}=V_{0}\cdot\delta+\frac{V_{0}^{2}}{2\alpha_{-}}$$

where: $\delta$ is the reaction time delay; $\alpha_{-}$ is the vehicle deceleration.

If the driver decides to pass through the signalized intersection, the maximum distance to pass through safety is[6]:

$$X_{c}=V_{0}\cdot\delta+V_{0}\cdot\left( \tau-\delta\right)+\frac{1}{2}\alpha_{+}\cdot\left( \tau-\delta\right)^{2}-\left( w+l \right)$$

where: $\tau$ is the rest of yellow signal time; $\alpha_{+}$ is the vehicle acceleration; $w$ is the width of intersection; $l$ is the length of vehicle.

If $X_{c}<X_{0}$, and the location of vehicle $X\in\left( X_{c},X_{0} \right)$ or If $X_{c}>X_{0}$, and the location of vehicle $X\in\left( X_{0}{,X}_{c} \right)$. The vehicle is in the dilemma zone as shown in Figs 4 and 5.

**Fig 4**. Type I dilemma zone

**Fig 5**. Type II dilemma zone

Traffic light is defined as a target object and combined with ranging sensors in CarSim. Roads and reference paths are created with Scene Builder and defined with configurable functions to build dilemma zone scenarios and traffic event sequences. The snapshot of traffic scene in simulator is showed as Figure 3.

**Figure 3.** The snapshot of traffic scene

We added this explanation into the simulation and model verification section as:

The range and location of dilemma zone for different driver is different. To make the trajectory data compareable, the observation range is setting as 100 meter along the driving approaching. The starting observation location is set at 100 meter before the stop-line for the vehicles which decide to stop before the stop-line (group 1). For vehicles which decide to go through the signalized intersection, the starting observation location is set at 50 meter before the stop-line (group 2).

**Comment #4:** Why only three car models are used in the simulation? To represent the driver behavior model more accurately and then potentially improving the understanding of the driver-vehicle-environment system in the dilemma zone, are more car models needed to be considered?

Response:

Thank you for your comments. We are sorry for not stating clearly about the vehicle model used in this paper.

CarSim includes example datasets for 10 vehicle types (with about 80 variants in total). Furthermore, users can create and define new vehicle model. The three vehicle models, A-Class (Hatchback), B-Class (Hatchback), and D-Class (SUV) are nearest to the reality traffic flow in urban city in China. Therefore, we just selected the three vehicle models to simulate. More vehicle models in CarSim or user-defined vehicle models can be analyzed by the proposed model in this paper.

We added this explanation into the simulation and model verification section as:

In China, cars and SUVs make up the bulk of traffic flow in urban city. The Hatchback and SUV models in CarSim are closest to the actual vehicles running on the road. Therefore, we use the vehicle models of A-Class (Hatchback), B-Class (Hatchback), and D-Class (SUV) in the CarSim software package to verify the proposed model in this paper.

**Response to Reviewer #2:**

This paper proposed a framework of driver behavior (perception, decision-making, and operation) using a stochastic predictive modeling method.

**Comment #1:** Recommend the authors adding several latest references in literature review.

Response:

Thank you for your comments and suggestions. We have added some latest references in the literature review. The latest references are:

7.	Pathivada BK, Vedagiri P. Investigating dilemma zone boundaries for mixed traffic conditions using support vector machines. Transp Lett. 2021. doi: 10.1080/19427867.2020.1870307.

8.	Pawar DS, Pathak D, Patil GR. Modeling dynamic distribution of dilemma zone at signalized intersections for developing world traffic. Journal of Transportation Safety & Security. 2020. doi: 10.1080/19439962.2020.1852464.

9.	Kang M-W, Rahman M, Lee J. Determination and Utilization of Dilemma Zone Length and Location for Safety Assessment of Rural High-Speed Signalized Intersections. Transportation Research Record. 2020;2674(4):272-80. doi: 10.1177/0361198120911929.

21.	Dong S, Zhou JB. A Comparative Study on Drivers' Stop/Go Behavior at Signalized Intersections Based on Decision Tree Classification Model. Journal of Advanced Transportation. 2020;2020. doi: Artn 125082710.1155/2020/1250827..

22.	Li J, Jiang B, Dong C, Wang J, Zhang X. Analysis of Driver Decisions at the Onset of Yellow at Signalized Intersections. Journal of Advanced Transportation. 2020;2020. doi: 10.1155/2020/2023093.

23.	Li J, Zhang H, Zhang Y, Zhang X. Modeling Drivers' Stopping Behaviors during Yellow Intervals at Intersections considering Group Heterogeneity. Journal of Advanced Transportation. 2020;2020. doi: 10.1155/2020/8818496.

24.	Najmi A, Choupani A-A, Aghayan I. Characterizing driver behavior in dilemma zones at signalized roundabouts. Transport Res F-Traf. 2019;63:204-15. doi: 10.1016/j.trf.2019.04.007.

**Comment #2:** The resolution of Fig. 6-18 needs to be improved.

Response:

Thank you for your comments and suggestions. We have improved the Figs’ resolution. The updated Figs are shown as:

**Fig 6**. The vehicle driving trajectory of different vehicle models in $N_{E}=10$ (Group 1).

**Fig 7**. The B-Class vehicle driving trajectory for different $N_{E}$ (Group 1).

**Fig 8**. The vehicle driving trajectory of different vehicle models in $N_{E}=10$ (Group 2).

**Fig 9**. The B-Class vehicle driving trajectory for different $N_{E}$ (Group 2).

|  |  |
| --- | --- |
| (a) changing of speed | (b) changing of acceleration |

**Fig 10.** The changes in vehicle speed and acceleration during the reactions at initial speed=30 km/h with different vehicle models (Group 1).

|  |  |
| --- | --- |
| (a) changing of speed | (b) changing of acceleration |

**Fig 11**. The changes in vehicle speed and acceleration during the reactions at initial speed=45 km/h with different vehicle models (Group 1).

|  |  |
| --- | --- |
| (a) changing of speed | (b) changing of acceleration |

**Fig 12**. The changes in vehicle speed and acceleration during the reactions at initial speed=70 km/h in different vehicle models (Group 1).

|  |  |
| --- | --- |
| (a) changing of speed | (b) changing of acceleration |

**Fig 13**. The changes in vehicle speed and acceleration during the reactions at initial speed=30 km/h with different vehicle models (Group 2).

|  |  |
| --- | --- |
| (a) changing of speed | (b) changing of acceleration |

**Fig 14**. The changes in vehicle speed and acceleration during the reactions at initial speed=45 km/h with different vehicle models (Group 2).

|  |  |
| --- | --- |
| (a) changing of speed | (b) changing of acceleration |

**Fig 15**. The changes in vehicle speed and acceleration during the reactions at initial speed=70 km/h with different vehicle models (Group 2).

|  |  |
| --- | --- |
| (a) group 1 | (b) group 2 |

**Fig 16**. Comparison diagram of operation time delay in different vehicle models at initial speed=30 km/h.

|  |  |
| --- | --- |
| (a) group 1 | (b) group 2 |

**Fig 17**. Comparison diagram of operation time delay in different vehicle models at initial speed=45 km/h.

|  |  |
| --- | --- |
| (a) group 1 | (b) group 2 |

**Fig 18**. Comparison diagram of operation time delay in different vehicle models at initial speed=70 km/h.

**Comment #3:** I suggest using real field data to validate at least one of the scenarios from the simulation cases and seeing if the conclusions still valid.

Response:

Thank you for your comments and suggestions. We quite agree with you to use real field data to validate the simulation cases and to verify the proposed model. Actually, we have used on-board GPS device to collect the trajectory data of vehicles when they approach a signalized intersection at the onset of yellow. However, the data-collection interval exceeds 10s and the location precision of GPS data is higher than 1meter. Therefore, the GPS trajectory data cannot be used to verify the high-fidelity vehicle dynamics models, which the data must be more high-resolution.

We just start to use roadside LiDAR to collect vehicles trajectory data in signalized intersection, which can provide trajectory data in millisecond-level and in centimeter-level accuracy theoretically. Currently, we focus on extracting high-resolution and trajectory-level traffic flow data in signalized intersection by roadside LiDAR. We attempt to collect the high-resolution and trajectory-level data of vehicles when they approach a signalized intersection at the onset of yellow by roadside LiDAR. We will use the field data to verify the proposed model combined with the high-resolution microscope traffic flow data extraction from roadside LiDAR in next paper.

**Comment #4:** Compared with state-of-art prediction methods, what is the strength of the proposed model?

Response:

Thank you for your comments. We are sorry for not stating our study clearly in the original submission. We would like to elaborate our contributions here again and also enhance the Conclusions part to the strength of the proposed model in the revised paper:

(1) A mathematical modeling method is used to study driver behavior in the dilemma zone based on stochastic model predictive control, along with considering the dynamic characteristics of human cognition and execution.

(2) Providing a feasible solution for modeling driver behavior more accurately and then potentially improving the understanding of the driver-vehicle-environment system in the dilemma zone, exploring the modeling framework of driver behavior in the dilemma zone, including the perception module, decision-making module, and operation module.

(3) We propose an SMPC-based high-fidelity vehicle dynamics and motion model to describe the processes of driver behavior at the onset of yellow signal indication when approaching the signalized intersection and use the CarSim simulator to verify the validity of the proposed model.

We added the strength of the proposed model into the Conclusions part as:

Based on the SMPC-based high-fidelity dynamics and motion model, the driver behavor can be reflected and estimated by identifying the changes of vehicle motion. In addition, the disturbances and uncertainties casued by internal factors or by external factors during driver approaches the signalized intersction at onset of yellow can be considered by adjust the parameter value of $N_{E}$ and $t_{d}\left( i \right)$ in dynamics formula.

**Comment #5:** The vehicles simulated in this study are only three models. Can more vehicle types be analyzed?

Response:

Thank you for your comments. We are sorry for not stating clearly about the vehicle model used in this paper.

CarSim includes example datasets for 10 vehicle types (with about 80 variants in total). Furthermore, users can create and define new vehicle model. The three vehicle models, A-Class (Hatchback), B-Class (Hatchback), and D-Class (SUV) are nearest to the reality traffic flow in urban city in China. Therefore, we just selected the three vehicle models to simulate. More vehicle models in CarSim or user-defined vehicle models can be analyzed by the proposed model in this paper.

We added this explanation into the simulation and model verification section as:

In China, cars and SUVs make up the bulk of traffic flow in urban city. The Hatchback and SUV models in CarSim are closest to the actual vehicles running on the road. Therefore, we use the vehicle models of A-Class (Hatchback), B-Class (Hatchback), and D-Class (SUV) in the CarSim software package to verify the proposed model in this paper.

**Comment #6:** Suggest more statistical analysis between the simulated results.

Response:

Thank you for your comments and suggestions. We found they are very useful and constructive. I have added more statistical analysis in the revision version.

For group 1, the value deceleration rate begin to change around the location of 50m before the stop-line. It can conclude that the dilemma zone is begin around at 50m before the stop-line for the vehicle driving at 45km/h of initial velocity. Higher value of $N_{E}$ means driver palys more attention on traffic condition when driving. From the trajectory data, the less value of $N_{E}$, the fluctuation of deceleration rate is more pronounced and deviates the expection of driver greater. $N_{E}=10$ is enough for driver to keep decelerating the vehicle safety. Furethermore, it can conclude that if the drivers concentrate enough on the traffic condition when driving, the trajectories of vehicle are similarity, and consist with the expectation of driver.

For group 2, the value acceleration rate increases continuously around the location of 50m before the stop-line. It can identify that the dilemma zone forming around 50m before the stop-line for the vehicle driving at 45km/h of initial velocity. It can conclude that if the drivers concentrate enough on the traffic condition when driving, the trajectories of vehicle are similarity, and consist with the expectation of driver. However, when passing through the signalized intersection, the trajectories deviation shows out in different vehicle model, especially in the driver with lower $N_{E}$ value.

For group 1, it shows out that the driver stepped on the brake pedal hastily first and then gradually smooth. The higher initial speed of vehicle, the driver will step on the brake pedal deeper and more haste. The lower initial speed of vehicle, the driver has more time to stop the vehicle smoothly before the stop-line. In addition, the different vehicle models show out different acceleration performance.

For group 2, it shows out that the driver stepped on the gas pedal gradually but with different strength. The lower initial speed of vehicle, the driver will step on the gas pedal deeper. The lower initial speed of vehicle, the driver need more time to accelerate the vehicle to go through the signalized intersection. And the driver will step on the gas pedal deeper to obtain more acceleration performance. However, the changing of speed in different initial speed seems similarity.
